# Optimizing Friction Stir Welding of Dissimilar Grades of Aluminum Alloy Using WASPAS

**DOI:** 10.3390/ma15051715

**Published:** 2022-02-24

**Authors:** Pinnavasal Venukrishnan Rajesh, Krishna Kumar Gupta, Robert Čep, Manickam Ramachandran, Karel Kouřil, Kanak Kalita

**Affiliations:** 1 Department of Mechanical Engineering, Saranathan College of Engineering, Trichy 620012, India; rajesh-mech@saranathan.ac.in; 2 Department of Mechanical Engineering, Malwa Institute of Science and Technology, Indore 453111, India; krishna.k.gupta@gmail.com; 3 Department of Machining, Assembly and Engineering Metrology, Faculty of Mechanical Engineering, VSB-Technical University of Ostrava, 708 00 Ostrava, Czech Republic; 4 Data Analytics Lab, REST Labs, Kaveripattinam 635112, India; ramachandran.manickam@restlabs.in; 5 Institute of Manufacturing Technology, Faculty of Mechanical Engineering, Brno University of Technology, Technicka 2896/2, 616 69 Brno, Czech Republic; karel.kouril@vutbr.cz; 6 Department of Mechanical Engineering, Vel Tech Rangarajan Dr. Sagunthala R&D Institute of Science and Technology, Avadi 600062, India

**Keywords:** aluminum, alloys, composites, stir casting, friction stir welding, parameters, properties, multi-attribute decision making, optimization

## Abstract

Aluminum is a widely popular material due to its low cost, low weight, good formability and capability to be machined easily. When a non-metal such as ceramic is added to aluminum alloy, it forms a composite. Metal Matrix Composites (MMCs) are emerging as alternatives to conventional metals due to their ability to withstand heavy load, excellent resistance to corrosion and wear, and comparatively high hardness and toughness. Aluminum Matrix Composites (AMCs), the most popular category in MMCs, have innumerable applications in various fields such as scientific research, structural, automobile, marine, aerospace, domestic and construction. Their attractive properties such as high strength-to-weight ratio, high hardness, high impact strength and superior tribological behavior enable them to be used in automobile components, aviation structures and parts of ships. Thus, in this research work an attempt has been made to fabricate Aluminum Alloys and Aluminum Matrix Composites (AMCs) using the popular synthesis technique called stir casting and join them by friction stir welding (FSW). Dissimilar grades of aluminum alloy, i.e., Al 6061 and Al 1100, are used for the experimental work. Alumina and Silicon Carbide are used as reinforcement with the aluminum matrix. Mechanical and corrosion properties are experimentally evaluated. The FSW process is analyzed by experimentally comparing the welded alloys and welded composites. Finally, the best suitable FSW combination is selected with the help of a Multi-Attribute Decision Making (MADM)-based numerical optimization technique called Weighted Aggregated Sum Product Assessment (WASPAS).

## 1. Introduction

An alloy is a mixture of two or more metals at specific proportions. Generally, all metals exist in the form of alloys. Aluminum, the most abundant metal in the Earth’s crust, is extracted from bauxite ore and purified as an alloy. Aluminum alloys contain aluminum as the major constituent (>90%), with minor traces of other metals. Based on the chemical composition (i.e., predominance of one or two minor constituents other than aluminum), aluminum alloys are categorized into different grades [1]. Each grade finds its usage in a particular application. These alloys can be made into blooms, billets or rods for commercial purposes, either by means of extrusion or casting.

Aluminum Matrix Composites (AMC) are formed when one or more non-metal (most preferably ceramics) is reinforced into the aluminum alloy, so that the resultant material will be having improved physical, mechanical and tribological properties, compared to individual alloy [2]. Even though AMCs are fabricated by numerous processing techniques, casting remains the most popular and inexpensive method.

Weldability is an important property that enables alloys or MMCs to be welded into different shapes and dimensions. The excellent weldability of aluminum makes it one of the most sought-after metal alloys for structural applications [3]. Welding is a metal joining process in which two metal pieces of regular cross-section are joined together with the application of heat. Friction Stir Welding is one of the recent techniques to join aluminum alloys and their associated composites [4,5].

Multi-Criteria Decision Making (MCDM) is a quantitative analysis-based optimization methodology in which the best possible solution is selected from a set of alternatives based on ranking, when contrasting criteria are taken into account [6]. It is broadly classified into two distinctive categories: one is Multi-Attribute Decision Making (MADM), a model to solve problems with a discrete data set, and the other is Multi-Objective Decision Making (MODM), a model to solve problems with a continuous or analog data set [7,8].

In this paper, MADM is used to identify the optimal combination of the FSW process as the results obtained from various tests are non-continuous/discrete in nature. By reviewing various literatures related to the FSW of aluminum alloys and AMCs, an inference has been obtained that FSW is done only in alloy–alloy combinations (similar or dissimilar grades) and composite–composite combinations, with the same ceramic used in both plates. To address this gap, an attempt has been made to fabricate and analyze FSW in alloy–composite combinations and composite–composite combinations, with different ceramics in both the side plates. The dissimilar aluminum alloys and composites are welded by FSW and experimentally characterized to find the ultimate tensile strength and hardness. The corrosion resistances of the welds are also experimentally analyzed. The rest of the paper is arranged as follows: Section 2 describes the experimental methodology used to fabricate the composites and their processing. Section 3 details the experimental findings in a systematic way. The findings of both the mechanical and corrosion tests are detailed here and carefully analyzed. Section 4 introduces and solves the multi-criteria decision-making problem in a step-by-step way. Finally, succinct conclusions based on the experimental and MCDM simulations are drawn in Section 5.

## 2. Methodology

### 2.1. Selection of Materials

For fabricating AMCs, the preliminary requirement is to select materials for matrix as well as reinforcement. This section of the paper deals with the selection and procurement of raw materials for casting.

(a) Matrices

Matrix is the encompassing part that covers the composite structure. In an AMC, it is necessarily an aluminum alloy with maximum proportion. Two distinctive grades of aluminum alloy Al 6061 and Al 1100 are selected as matrices.

Al 6061 is the most common and abundantly used precipitation-strengthened wrought aluminum alloy with significant percentages of silicon and magnesium. It has good workability, superior surface finish and excellent corrosion resistance [1]. The proportion of metals present in Al 6061 alloy is given below in Table 1.

Al 1100 is the purest alloy in the wrought aluminum family of 1xxx series. This mechanically strongest alloy has good thermal properties and better workability. The chemical composition of Al 1100 is given below in Table 2.

(b) Reinforcements

Reinforcement is the material present at minimum proportion that is inserted in the metallic matrix as a fiber, powder or whisker. It provides binding strength to the composite therein. Two popular ceramics, Alumina (Al_2_O_3_) and Silicon Carbide (SiC), are selected as reinforcements in this research work. These ceramics are synthetic in nature, with good abrasive properties and heat-withstanding capacity. They are used to increase the hardness and wear resistance of the composite [2,9]. The general properties of both the ceramics are given in Table 3.

### 2.2. Fabrication Method

The method used to fabricate composite specimens and sometimes alloys of unusual metal-mix ratio is stir casting, alternatively known as gravity die casting [10]. In this method, the metal alloy is melted and casted into required shape and size. Initially, the metal alloys to be melted are put into graphite crucible and kept inside a coal-ignited furnace. The combustion of the furnace is controlled using a mechanized blower. When the aluminum alloys reach liquidus stage or semi-solid state, the preheated ceramic powders are poured inside the molten metal and stirred thoroughly (either manually or with the help of propeller) to ensure uniform distribution of reinforced particles inside metallic matrix. Insufficient stirring may result in the stagnation of ceramic powders as residue at the bottom of crucible. This mixture is then poured into mould cavity of desirable shape and dimension [1]. In present research work, it is a mild steel die. After solidification, the casted composite is removed from the die and quenched in normal air. Weight of metal alloy required for casting 1 composite plate measuring 150 × 150 × 5 mm^3^ as per mass-based ratio is as follows:

Density of Al 6061 = Al 1100 ≈ 2.7 g/cm^3^;

Required Volume of the composite = 15 × 15 × 0.5 = 112.5 cm^3^;

Density = mass/volume;

Required mass of the metal alloy = density × volume = 2.7 × 112.5 = 303.75 g;

Casting allowance (shrinkage allowance) = 15% of mass;

Hence, final required mass of metal = 303.75 + (0.15 × 303.75) = 349.31 g ≈ 350 g.

In mass-based ratio, the percentage of metal alloy is kept constant as 100%. Increase in metal alloy % will increase the dimension or size of the composite. For required shape to be obtained, proportion of metal alloy should not be reduced as in volume-based ratio. Reduction in metal alloy % will result in incomplete shape of casting. Addition of reinforcement powders will help to increase weight of the composite as both the ceramics are denser than the two different grades of aluminum alloy. The alloys and composites’ combination fabricated using stir casting is mentioned below. Three specimens are fabricated for each composition.

1. 100% Al 6061 (350 g);
2. 100% Al 1100 (350 g);
3. 100% Al 6061 (350 g) + 5% Al_2_O_3_ (17.5 g);
4. 100% Al 1100 (350 g) + 5% Al_2_O_3_ (17.5 g);
5. 100% Al 6061 (350 g) + 5% SiC (17.5 g);
6. 100% Al 1100 (350 g) + 5% SiC (17.5 g).

### 2.3. Processing Technique

**Figure 1:**
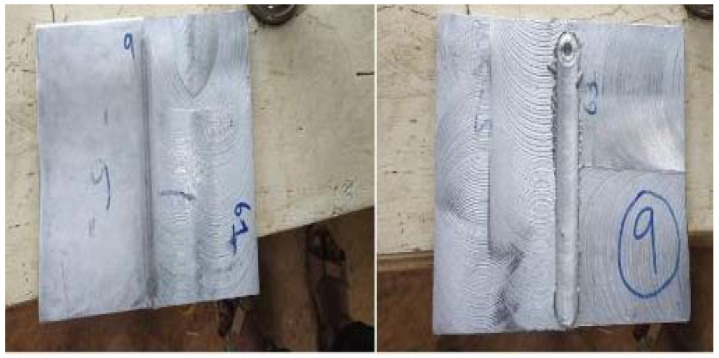
Friction Stir Welding specimens.

This section deals with friction stir welding of composite plates. FSW is the predominantly used technique to join two plates, made up of aluminum alloys or aluminum composites [11]. The composite or alloy plates to be welded together are placed in close proximity, so that their longitudinal edges touch each other and are hinged. The entire work-holding setup is placed in vertical milling machine (Precicut, Noida, India). The tool holder is comprised of a cylindrical tool made of stainless steel of diameter 15 mm with nose tip radius and height measuring 3 mm and 4 mm, respectively. When the rotating tool comes into contact with the stationary plates, a welding action is created throughout the meeting line (junction) of the two plates, due to heat generated by friction thereby joining them [5,12]. The process parameters used in FSW are presented in Table 4. Figure 1 shows a set of plates joined by FSW.

The 9 different specimen combinations created by FSW are represented as follows:

1. Al 6061 with Al 1100;
2. Al 6061 with Al 1100/5% Al_2_O_3;_
3. Al 6061 with Al 1100/5% SiC;
4. Al 1100 with Al 6061/5% Al_2_O_3;_
5. Al 1100 with Al 6061/5% SiC;
6. Al 6061/5% Al_2_O_3_ with Al 1100/5% Al_2_O_3;_
7. Al 6061/5% Al_2_O_3_ with Al 1100/5% SiC;
8. Al 6061/5% SiC with Al 1100/5% Al_2_O_3;_
9. Al 6061/5% SiC with Al 1100/5% SiC.

It should be noted that the FSW process parameters have significant influence on the mechanical and morphological properties of the weld. Thus, optimizing them is also a realistic goal. However, optimization of FSW process parameters for each of the nine material combinations listed above would require exhaustive experimentation. Even by using design of experimentation methods such as Box–Behnken, central composite design, etc., the number of experiments needed for all the material combinations to ascertain the optimal process parameters will be very large. Thus, in this study the process parameters in Table 4 are decided through an extensive literature review.

## 3. Experimental Results

Evaluation of FSW process is done through conducting various tests that depict different material behavior. In this research work, appropriate tests are conducted to analyze the mechanical properties such as tensile strength and hardness, as well as corrosion resistance behavior of the different weld combinations that consist of alloys and composites. Values obtained from mechanical tests and corrosion tests are also compared among the weld specimens to ascertain the corresponding tendency (as to which combination gives best results).

### 3.1. Mechanical Tests

The tests covered under this section are performed to identify the ultimate tensile strength (UTS) and Brinell Hardness Number (BHN) of the weld specimens. The test samples are prepared with dimensions as per ASTM Standard Test Methods.

#### 3.1.1. Tensile Test

**Figure 2:**
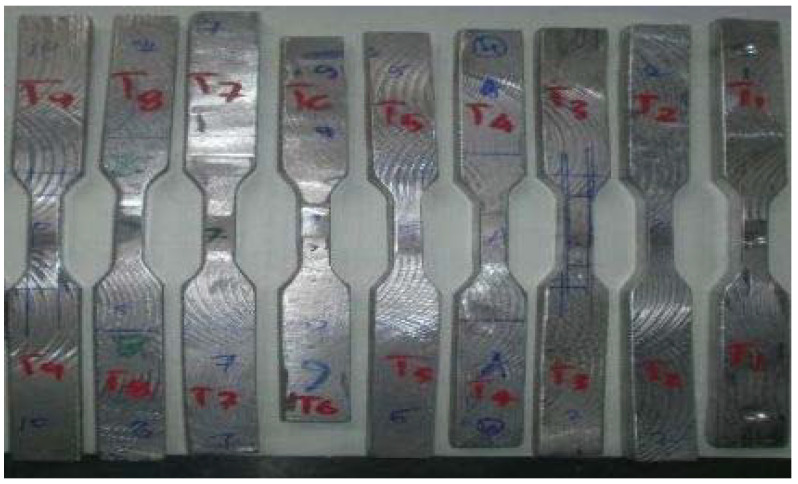
Tensile test samples as per ASTM Standards.

This test is done to measure the ductility of the material. Ductility is the tendency, which makes the material more flexible so that it can be drawn into lengthy and thin wire [1]. Ductile materials such as aluminum are more elastic in nature. Tensile test is conducted in tension chamber (Blue Star, Mumbai, India) of universal testing machine (UTM). The FSW specimens of different combinations are cut and prepared as shown in Figure 2. These test samples are fixed between a fixed jaw and moving jaw of UTM one after the other. When the movable jaw starts to move vertically due to hydraulic power, pulling force is applied to the sample and starts to elongate. As the sample enters the plastic region after leaving elastic region, it absorbs maximum stress before breaking. Maximum change in length (increase in length and reduction in cross-sectional area) occurs during the exertion of maximum load or ultimate tensile stress. Table 5 represents the results of tensile test conducted on 9 FSW combination samples.

The results indicate that the FSW specimen 4 in which Al 1100 alloy is welded with Al 6061/5% Al_2_O_3_ composite gives the highest UTS. This indicates perfect bond between two plates at weldment region. The FSW combination of Al 6061 alloy with Al 1100 alloy gives the second highest value compared to other combinations, as alloy exhibits more ductility than composite. The welding combination in which Al 6061/5% SiC is joined with Al 1100/5% Al_2_O_3_ has lowest tensile strength as the presence of both ceramics induces brittleness and makes the FSW bond weak. The FSW combinations involving SiC produce reduced tensile strength as SiC is harder than Al_2_O_3_.

#### 3.1.2. Hardness Test

This test is done to measure the hardness of the specimens by calculating Brinell Hardness Number (BHN). Hardness is the extent to which a material can withstand abrading or indenting load. The specimens are placed flat in the hardness tester so that a point load of 250 kgf is applied over the weldment surface by a steel ball indenter of diameter 5 mm and held constantly for 10 s. The diameter of the impression created by indenter is inversely proportional to the hardness [9]. The BHN is calculated from the following Equation (1). The Brinell hardness values of the 9 FSW specimens are tabulated below in Table 6.

(1) $$\mathrm{BHN}\;=\frac{2P}{\pi D\left[D-\sqrt{D^{2}-d^{2}}\;\right]}$$

where

*P* = constant load applied on weldment (250 kgf);

*D* = diameter of steel ball indenter (5 mm);

*d* = diameter of the impression in mm.

**Table 6:** Hardness test results.

Specimen No.	Diameter of the Impression, d	Brinell Hardness
	Mm	BHN
1	3.80	42.43
2	3.67	45.61
3	3.50	50.34
4	3.57	48.30
5	3.47	51.23
6	3.53	49.44
7	3.40	53.43
8	3.43	52.47
9	3.33	55.77

The results indicate that friction-stir-welded AMCs are having higher hardness than friction-stir-welded aluminum alloy. The addition of SiC plays a significant role as it provides more hardness than Al_2_O_3_. The alloy weld combination has the minimum hardness, whereas the SiC-reinforced composite weld combination shows maximum hardness. The alloy–composite weld specimens are harder than alloy–alloy weld specimens but have lower hardness value than composite–composite weld specimens.

### 3.2. Corrosion Test

Corrosion is a bulk material removal process. It is defined as the removal of material throughout the specimen, due to some chemical action or reaction. Corrosion test is done to identify the specimen, which is having best corrosion resistance among different combinations. Immersion corrosion test is the most popular and widely used corrosion test, especially for aluminum alloys and AMCs [9]. In this test, samples are cut from the nine weld combinations such that the weldment region lies in between the sample and is prepared as per ASTM Standard G31. These samples are immersed separately in acid and alkaline (base) solutions of known concentration (1 normality) for a specific period of time (24 h). Then, they are taken out and the mass loss due to corrosion is found out from the difference between the initial weight of the sample before dipping it into corroding medium and the final weight of the sample after removing it from the corroding medium. Finally, the corrosion rate is calculated from the formula mentioned in Equation (2):

(2) $$CR=\frac{87.6W}{\rho AT}$$

where

*CR* = corrosion rate expressed in millimetre per year (mm/y);

*W* = mass loss due to corrosion in mg;

*ρ* = density of the sample in g/cm^3^;

*A* = cross-sectional area of the sample in cm^2^;

*T* = time of exposure in hours.

Then, the unit of corrosion rate is converted into mils per year (mpy) using the below mentioned Equation (3).

(3) 1 mpy =0.0254 mm/y

Lower the corrosion rate, better the corrosion resisting capacity of the weld specimen. Table 7 represents the corrosion rate of the specimens immersed in acidic and basic media.

The inferences obtained from the above test results clearly show that the alloy–composite and composite–composite weld combinations offer more corrosion resistance than alloy–alloy weld combination. The composite–composite weld combinations fare even better than alloy–composite combination. The reinforcement of alumina plays a crucial role in reducing the corrosion rate in weld specimens. The specimens containing alumina have better corrosion resistance than the specimens with Silicon Carbide.

## 4. Selection of Optimal FSW Combination Using Weighted Aggregated Sum Product Assessment (WASPAS) 

The weighted aggregated sum product assessment (WASPAS) is one of the most recent MADM-inspired optimization approaches, which uses a unique combination of weighted sum model (WSM) and weighted product model (WPM) to induce higher ranking accuracy [13]. This method was first proposed by Zavadskas et al. in 2012 [14]. In this method, the relative importance of each attribute/criterion is determined initially followed by the evaluation and prioritization of the given set of alternatives.

The formulae and the step-by-step procedure for optimizing the above detailed FSW process is given below.

(a) Decision Matrix Table

The optimization process starts with the construction of decision matrix table. Here, ‘*m*’ refers to the alternatives (i.e., nine FSW combinations) and ‘*n*’ denotes attributes or criteria (i.e., four output properties). Rows are denoted by ‘*i*’, and columns are denoted by ‘*j*’. *X_ij_* refers to the *j*th attribute value of *i*th alternative [15]. The decision matrix is represented as Equation (4).

(4) $$D=\left[\begin{array}{ccc} X_{11} & \cdots & X_{1n}\\ \vdots & \ddots & \vdots \\ X_{m1} & \cdots & X_{mn} \end{array}\right]$$

Table 8 indicates the decision matrix table created for the above-explained research work.

(b) Linear Normalization of Decision Matrix

Normalization of decision matrix table elements is done to convert those values into same unit factors [15]. The formulae for normalizing elements in beneficiary and non-beneficiary attributes are given as Equations (5) and (6).

For beneficiary attribute,

(5) $$r_{ij}=\frac{x_{ij}}{\underset{i}{\max}x_{ij}}$$

For non-beneficiary attribute,

(6) $$r_{ij}=\frac{\underset{i}{\min}x_{ij}}{x_{ij}}$$

Table 9 indicates normalized decision matrix.

Table 10 indicates the attributes that fall under beneficiary and non-beneficiary category, with the corresponding weight factors based on their relative importance.

(c) Creation of joint criterion of optimality

First criterion of optimality deals with weighted sum model (WSM)-based calculation of relative importance of *i*th alternative using the following Equation (7),

(7) $$Q_{i\left(1\right)}=\sum_{j=1}^{n}\left(r_{ij}*w_{j}\right)$$

**Figure 3:**
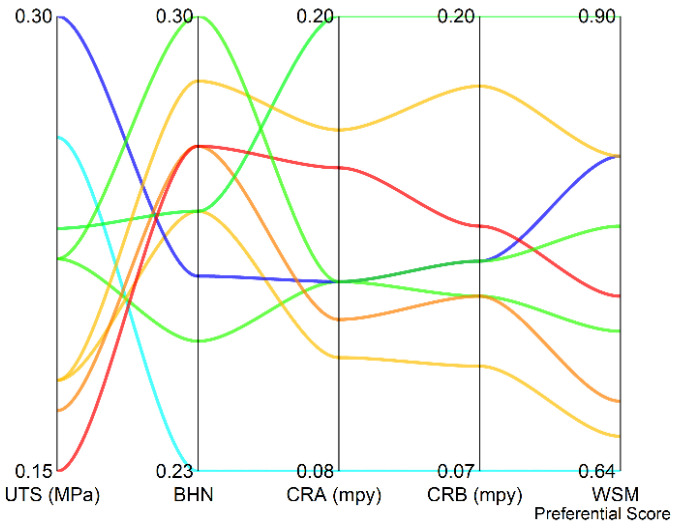
Parallel plot denoting the weighted vectors and weighted sum model (WSM) preferential score.

Figure 3 shows the weighted normalized decision matrix along with the WSM preferential score. Since the UTS and BHN are maximization type whereas the CR_A_ and CR_B_ are minimization type, the parallel plot provides a good visualization tool to inspect the effect of each of the four responses on the WSM preferential score. It should be noted that due to Equations (5) and (6), the minimization type responses are on an inverted scale with reference to the maximization type responses. It is observed from Figure 3 that the combination of a moderate level of UTS and BHN and least value of CR_A_ and CR_B_ is determined as the best solution as per WSM.

Second criterion of optimality deals with weighted product model (WPM)-based calculation of relative importance of *i*th alternative using the following Equation (8):

(8) $$Q_{i\left(2\right)}=\overset{n}{\underset{j=1}{\prod }}{\left(r_{ij}\right)}^{w_{j}}$$

**Figure 4:**
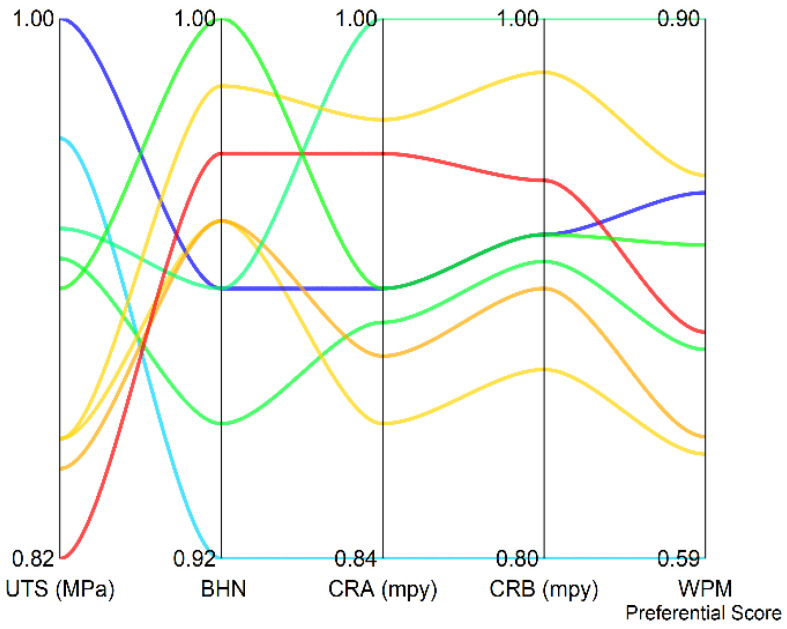
Parallel plot denoting the weighted vectors and weighted product model (WPM) preferential score.

Figure 4 shows the weighted normalized decision matrix along with the WPM preferential score. The behavior of the weighted vectors of the responses for WPM is also seen to be similar to WSM. Here too, the poorest solution has relatively high UTS but the least BHN. The CR_A_ and CR_B_ are also the highest for this solution (i.e., specimen no. 1).

Joint criterion of optimality is created by a generalized equation Equation (9) with the aggregation of preferential scores obtained from additive and multiplicative methods, with ‘*λ*’ as a common factor of multiplicative constant [16]. For enhanced accuracy, *λ* is kept as 0.6.

(9) $$Q_{i}=\lambda Q_{i\left(1\right)}+\left(1-\lambda \right)Q_{i\left(2\right)}\;i.e.,\;Q_{i}=\lambda \prod_{j=1}^{n}\left(r_{ij}*w_{j}\right)+\left(1-\lambda \right)\sum_{j=1}^{n}r_{ij}{}^{w_{j}}$$

(d) Ranking order

The joint criterion of optimality, also known as WASPAS coefficient, which is used for ranking the alternatives in ascending order from best to worst along with the ranks [16,17] and corresponding alternatives/specimens, is listed in Table 11 below. The attribute values of the best possible/optimal FSW combination are tabulated below in Table 12. From the above Table, it is clear that the FSW specimen 6 that contains Al 6061/5% Al_2_O_3_ joined with Al 1100/5% Al_2_O_3_ is the best possible combination.

## 5. Conclusions

Aluminum alloys are increasingly finding numerous applications, where high strength-to-weight ratio, low weight, good surface finish and excellent thermal properties are required. Aluminum Matrix Composites are being predominantly explored by researchers due to their enhanced mechanical, chemical and tribological properties. The evaluation of mechanical properties and corrosion resistance by comparing the results can enable researchers to analyze the FSW process in terms of joining dissimilar AMCs and Al alloys. Based on a comprehensive experimental study, the following conclusions can be drawn.

- The UTS of Al 1000 alloy and Al 6061/5% Al_2_O_3_ was the highest among the tested samples, while for weldment of Al 6061/5% SiC and Al 1100/5% Al_2_O_3,_ the UTS was the lowest. This may be due to the presence of ceramics, which induces brittleness and weakens the joint.
- In general, from a UTS viewpoint, weld joints involving alumina-based AMCs were observed to be better than those involving Silicon-Carbide-based AMCs. This might be due to the higher hardness of Silicon-Carbide-based AMCs.
- The hardness of friction-stir-welded AMCs was observed to be better than the friction-stir-welded Al alloys. This is due to the presence of SiC and Al_2_O_3_.
- In terms of corrosion resistance, the welded joints can be ranked from best to worst as composite–composite welds > alloy–composite welds > alloy–alloy welds. AMCs with alumina proved better than Silicon Carbide AMCs.
- WASPAS was easy to implement, and it ranked the welding of Al 6061/5% Al_2_O_3_ and Al 1100/5% Al_2_O_3_ as the best compromise solution.

One of the limitations of the study was that only aluminum-based alloys and composites were considered in the study. The study can be further improved by considering the various FSW process parameters as well as the different compositions. Use of newer multi-criteria decision-making methods such as SECA and CoCoSo, and global optimization routines, will perhaps also augment the optimal solution.

## Figures and Tables

**Table 1:** Elemental analysis of Al 6061.

Constituents	Al	Mn	Fe	Cu	Mg	Si	Cr
Percentage	97.74	0.11	0.13	0.39	0.97	0.62	0.08

**Table 2:** Elemental analysis of Al 1100.

Constituents	Al	Mn	Fe	Cu	Mg	Si	Cr
Percentage	99.00	0.05	0.40	0.05	0.04	0.40	0.01

**Table 3:** General properties of the ceramics.

Properties	Units	Aluminum Oxide (Al_2_O_3_)	Silicon Carbide (SiC)
Density	g/cm^3^	3.98	3.1
Melting point	°C	2300	2730
Vickers hardness	-	1560	4483
Fracture toughness	MPa√m	4.9	4.6
Elastic modulus	GPa	300	410
Tensile strength	MPa	210	137.9
Thermal conductivity	W/Mk	21	120
Coefficient of thermal expansion	m/°C	9.0 × 10^−6^	4.0 × 10^−6^

**Table 4:** Process parameters in Friction Stir Welding.

Parameters	Values	Units
Tool rotational speed	1000	rpm
Traverse feed	50	mm/min
Direction of tool rotation	Clock-wise	-
Tool pin shape	Cylindrical	-
Tool material	Stainless steel	-
Tool pin nose radius	3	mm

**Table 5:** Tensile test results.

Specimen No.	Maximum Load Given to Specimen, P_max_	Original Cross-Section Area at Neck Region (bxt), A_o_	UTS, T = P_max_/A_o_	Elongation = Change in Length/Original Length
	N	mm^2^	Mpa	%
1	4790	39.92	119.99	6.82
2	4080	40.24	101.39	5.84
3	3320	39.97	83.06	6.06
4	5450	40.12	135.84	4.78
5	3100	39.88	77.73	5.24
6	4260	40.17	106.05	6.32
7	3330	40.08	83.08	6.48
8	2770	40.02	69.22	4.56
9	3920	40.22	97.46	5.68

**Table 7:** Immersion corrosion test results.

Specimen No.	Acidic Corrosion Rate, CR_A_		Basic Corrosion Rate, CR_B_	
	In Acid Solution (HCl)		In Base Solution (NaOH)	
	mm/y	mpy	mm/y	mpy
1	28.01	0.71	52.29	1.33
2	18.12	0.46	28.17	0.72
3	21.45	0.54	33.76	0.86
4	17.54	0.45	26.93	0.68
5	19.72	0.50	29.61	0.75
6	11.28	0.29	17.15	0.44
7	13.38	0.34	19.46	0.49
8	14.18	0.36	24.07	0.61
9	16.75	0.43	25.86	0.66

**Table 8:** Decision matrix.

Specimen No.	Attributes			
	UTS (MPa)	BHN	CR_A_ (mpy)	CR_B_ (mpy)
1	119.99	42.43	0.71	1.33
2	101.39	45.61	0.46	0.72
3	83.06	50.34	0.54	0.86
4	135.84	48.3	0.45	0.68
5	77.73	51.23	0.5	0.75
6	106.05	49.44	0.29	0.44
7	83.08	53.43	0.34	0.49
8	69.22	52.47	0.36	0.61
9	97.46	55.77	0.43	0.66

**Table 9:** Normalized decision matrix.

Specimen No.	Attributes			
	UTS (MPa)	BHN	CR_A_ (mpy)	CR_B_ (mpy)
1	0.88	0.76	0.41	0.33
2	0.75	0.82	0.63	0.61
3	0.61	0.90	0.54	0.51
4	1.00	0.87	0.64	0.65
5	0.57	0.92	0.58	0.59
6	0.78	0.89	1.00	1.00
7	0.61	0.96	0.85	0.90
8	0.51	0.94	0.81	0.72
9	0.72	1.00	0.67	0.67

**Table 10:** Attributes and their weightage.

Sl. No.	Criteria	Category	Objective	Weightage, w*_j_*
1	UTS	Beneficiary (Larger-the-better)	Maximization	0.30
2	BHN	Beneficiary (Larger-the-better)	Maximization	0.30
3	CR_A_	Cost (Smaller-the-better)	Minimization	0.20
4	CR_B_	Cost (Smaller-the-better)	Minimization	0.20

**Table 11:** Ranking of alternatives.

Specimen No.	WSM Preferential Score, Q*_i_*_(1)_	WPM Preferential Score, Q*_i_*_(2)_	WASPAS Coefficient, Q*_i_*	Rank
1	0.64	0.59	0.62	9
2	0.72	0.71	0.72	6
3	0.66	0.65	0.66	8
4	0.82	0.80	0.81	3
5	0.68	0.66	0.67	7
6	0.90	0.90	0.90	1
7	0.82	0.81	0.82	2
8	0.74	0.72	0.73	5
9	0.78	0.77	0.78	4

**Table 12:** Optimized FSW specimen.

WASPAS Coefficient, Q_i_	Rank	Specimen No.	UTS (MPa)	BHN	CR_A_ (mpy)	CR_B_ (mpy)
0.90	1	6	106.05	49.44	0.29	0.44

## Data Availability

The data presented in this study are available in the article.
